# Structural studies of neuropilin‐2 reveal a zinc ion binding site remote from the vascular endothelial growth factor binding pocket

**DOI:** 10.1111/febs.13711

**Published:** 2016-04-01

**Authors:** Yi‐Chun Isabella Tsai, Constantina Fotinou, Rohini Rana, Tamas Yelland, Paul Frankel, Ian Zachary, Snezana Djordjevic

**Affiliations:** ^1^ Institute of Structural and Molecular BiologyUniversity College LondonLondonUK; ^2^ Magnus Life ScienceUniversity College LondonLondonUK; ^3^ Centre for Cardiovascular Biology & MedicineBHF Laboratories at University College LondonLondonUK; ^4^ Present address: Francis Crick InstituteMill Hill LaboratoryThe RidgewayLondonNW7 1AAUK

**Keywords:** neuropilins, signalling, vascular endothelial growth factor, X‐ray crystallography, zinc

## Abstract

Neuropilin‐2 is a transmembrane receptor involved in lymphangiogenesis and neuronal development. In adults, neuropilin‐2 and its homologous protein neuropilin‐1 have been implicated in cancers and infection. Molecular determinants of the ligand selectivity of neuropilins are poorly understood. We have identified and structurally characterized a zinc ion binding site on human neuropilin‐2. The neuropilin‐2‐specific zinc ion binding site is located near the interface between domains b1 and b2 in the ectopic region of the protein, remote from the neuropilin binding site for its physiological ligand, i.e. vascular endothelial growth factor. We also present an X‐ray crystal structure of the neuropilin‐2 b1 domain in a complex with the C‐terminal sub‐domain of VEGF‐A. Zn^2+^ binding to neuropilin‐2 destabilizes the protein structure but this effect was counteracted by heparin, suggesting that modifications by glycans and zinc in the extracellular matrix may affect functional neuropilin‐2 ligand binding and signalling activity.

**Abbreviations**
HBD: heparin binding domain
NRP1: neuropilin‐1
NRP2: neuropilin‐2
VEGF: vascular endothelial growth factor

## Introduction

Neuropilin‐2 (NRP2) and neuropilin‐1 (NRP1) are homologous transmembrane receptors that are expressed in diverse tissues, sharing a common domain organization and 44% amino acid sequence identity in humans 1, 2, 3. Both NRPs have essential roles in the control of axonal homing in the developing nervous system, as revealed by the phenotypes of genetically deficient mice 4, 5, 6, 7. NRP1 is also required for embryonic cardiovascular development, whereas NRP2 has a more restricted developmental role in lymphangiogenesis 7. In addition, neuropilins have been implicated in other physiological and disease‐related processes in adult organisms, such as various cancers, proliferative retinopathies and immunomodulation 8.

Neuropilins mediate signalling by binding to vascular endothelial growth factors (VEGFs) and semaphorins, secreted ligands that are essential for cardiovascular and neuronal development, respectively 9, 10, 11, 12, 13, 14. The ligand specificities for NRPs are often found overlap in cell‐based studies, whereas NRP1 and NRP2 display distinct expression patterns and ligand preferences *in vivo* 6, 11, 15, 16. In the central nervous system, for example, the semaphorin Sema3A preferentially signals via NRP1 to control cortical neuron basal dendritic arborization, whereas Sema3F binds NRP2 to regulate formation of apical dendrite processes of cortical neurons 17. In the vascular system, the ligand preferences for NRPs are less clearly defined, but the VEGF‐A splice variant VEGF‐A_165_ appears to be a major ligand for NRP1 on endothelial cells, whereas VEGF‐C signals via NRP2 in lymphatic vessels 9, 11, 18, 19, 20. Furthermore, NRP2 is co‐expressed with NRP1 in endothelial and other cell types, NRP2 and NRP1 may associate to form heterodimers 14, and NRP2 also binds to VEGF‐A in trimeric complexes with VEGF receptor 2 21, 22, suggesting that NRPs have the potential to interact in a cooperative fashion in the endothelium.

The protein regions of VEGF‐A encoded by exons 2‐5 form a cysteine knot structure that interacts with VEGF receptors, while the 55 amino acid residue C‐terminal domain of VEGF‐A, encoded by exons 7 and 8, is required for binding to neuropilin and heparin 23, 24. Several structural and biochemical studies have addressed the molecular basis of the VEGF binding selectivity of neuropilins. In all of the available structures, including those of a small molecule peptidomimetic in complex with NRP1 and of a fusion of the NRP1 binding region of VEGF‐A with the NRP1 b1 domain, a key feature is binding of a C‐terminal arginine residue to a conserved region of the b1 domain of neuropilin 25, 26, 27. Crystal structures of a fusion of human NRP1 b1 (NRP1 residues 274–429) with the heparin binding domain (HBD) of VEGF‐A_165_ (VEGF‐A_165_‐HBD; VEGF‐A_165_ residues 115–165) 26 or of human NRP2 domains b1b2 (NRP2 residues 276–595) fused with the C‐terminal five amino acids of mature VEGF‐C (SIIRR, residues 219–223) 27 revealed a high degree of similarity with respect to the interactions of the C‐terminal arginine residue of VEGF‐A_165_ and VEGF‐C with NRP1 and NRP2, respectively. Initial site‐directed mutagenesis experiments addressing sequence differences in the binding sites of NRP1 and NRP2 have led to the proposal that a charge repulsion mechanism provides the molecular basis for ligand selectivity 26, 27. However, more comprehensive structural and functional studies of NRP2, including other members of the VEGF ligand family, are needed to fully characterize and understand the molecular basis for both VEGF binding to NRP2 and differences between NRP1 and NRP2 in terms of ligand preference and biological function.

We now report structural and biochemical characterization of a conserved Zn^2+^ ion binding site on NRP2. This binding site is not present in NRP1, and we hypothesize that it may be involved in modulation of ligand binding to NRP2. In addition, we describe the three‐dimensional structure of a molecular complex between the C‐terminal region of VEGF‐A_165_ and the b1 ligand binding domain of NRP2, further addressing the question of ligand selectivity.

## Results

### Crystal structure reveals a NRP2‐specific zinc ion binding site

**Figure 1:**
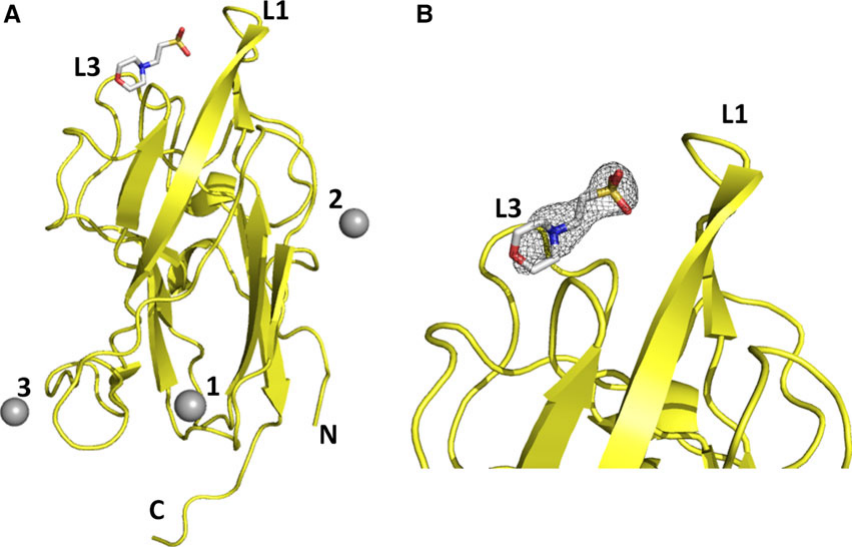
Zn^2+^ binding site on NRP2. (A) The X‐ray crystal structure of NRP2 crystallized in the presence of zinc revealed three zinc ions bound to the b1 domain. The structure is shown in yellow ribbon representation, and the three zinc ions are shown as grey spheres. A molecule of MES (2‐ethanesulfonic acid) bound to the canonical ligand binding site on the neuropilin b1 domain is shown using sticks representation. Loops L1 and L3 that shape the binding cleft are indicated. (B) Enlarged view of the ligand binding site, with the MES‐associated 2 *F*_o_ – *F*_c_ electron density map shown as a grey wire mesh and contoured at 1 σ.

**Figure 2:**
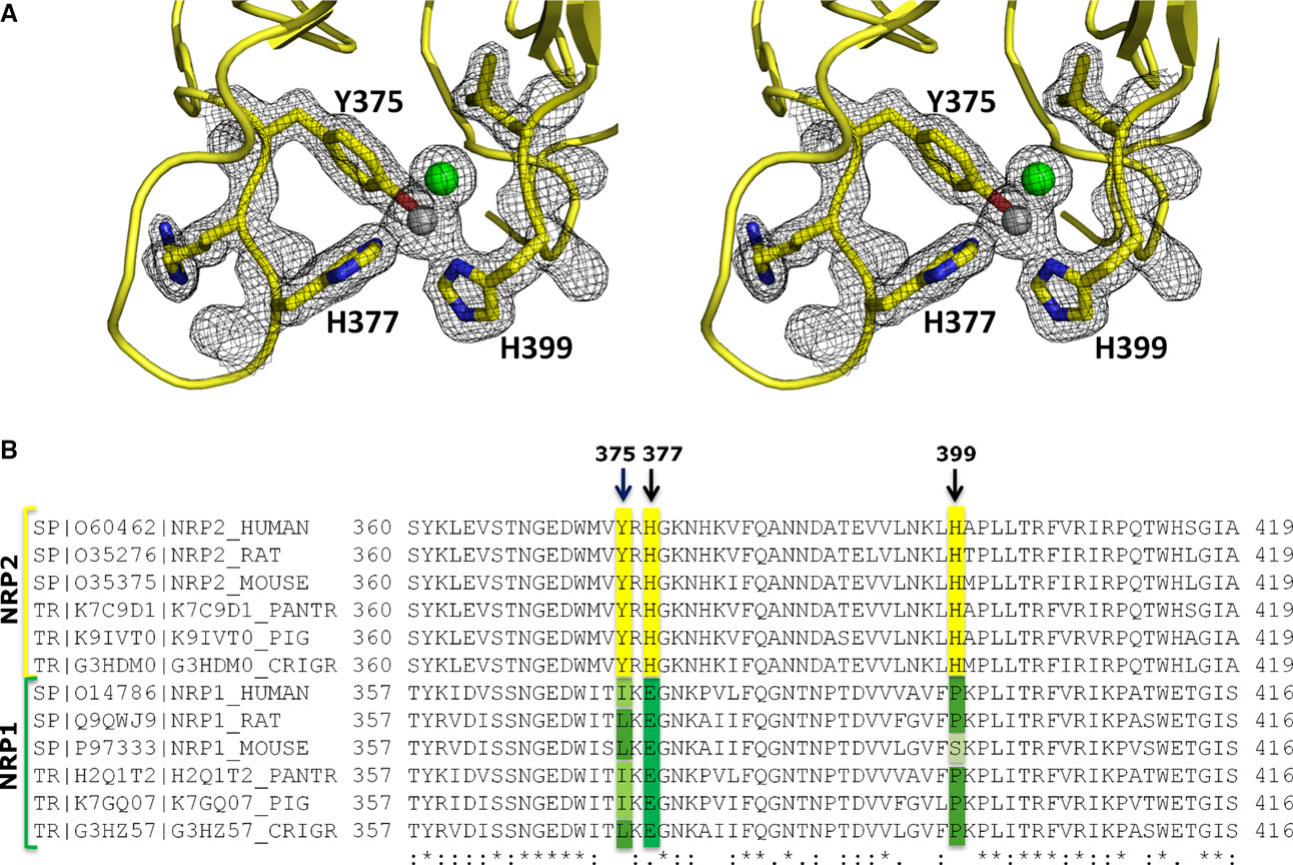
The main zinc ion binding site is NRP2‐specific. (A) Stereoimage of the NRP2 residues involved in Zn^2+^ ion coordination: Y375, H377 and H399. The residues are shown using stick representation within the final 2 *F*_o_ – *F*_c_ electron density map contoured at 1 σ (grey mesh). (B) Sequence alignment for the part of the neuropilin sequences that includes all three residues that bind zinc in site ‘1’ shows that the site is conserved in NRP2 and absent in NRP1. The residues associated with NRP2 are highlighted in yellow, and the residues in the analogous positions in NRP1 are highlighted in green.

We have solved a structure of NRP2 from the crystal grown in the presence of zinc sulfate that diffracted to 1.8 Å resolution (Fig. 1 and Table 1). No specific peptidic ligand was present during the crystallization, but the ligand binding pocket showed a difference electron density that was consistent with a molecule of MES buffer (2‐ethanesulfonic acid) present in the mother liquor, although with the significant disorder of the morpholine ring (Fig. 1). In addition, three zinc ion binding sites were identified in the difference electron density maps, and the location of zinc ions was verified by an anomalous difference map (Fig. 1A). The most significant zinc ion binding site (referred to as site ‘1’) is formed by the side chains of the amino acids Tyr375, His377 and His399, with the fourth zinc ion coordination most likely provided by a chloride ion from the protein buffer solution used for crystallization (Fig. 2A). We interpreted the density as representing a chloride ion, as when water molecule was modelled in that position, there was always strong residual electron density left. The other two zinc ions bound to NRP2 in this crystal structure are less likely to be biologically relevant, and their binding sites have probably been generated by the crystallization conditions and crystal packing. Both of the additional two binding sites are formed within the inter‐molecular space, with two zinc‐coordinating side chains provided by one molecule and the third side chain provided by an amino acid of the crystallographic neighbour. Specifically, the second zinc ion is coordinated by Glu284 and His312 from one molecule and His415 from the neighbouring molecule; the third zinc ion is coordinated by Lys362 and His381 from one molecule and Glu370 from the neighbouring molecule. The zinc‐coordinating residues at site 1 of NRP2 are conserved throughout mammalian species and unique to NRP2 as they are absent in NRP1 sequences (Fig. 2B), further suggesting a specific physiological role for this metal binding site. The orientation of the three coordinating side chains in the presence of zinc ion within our structure is different from that of the side chains of equivalent residues in the previously reported NRP2 structure 28, reflecting adjustment of the side chain conformations in order to accommodate ion binding.

**Table 1:** Data collection and refinement statistics. Values in parentheses are for the highest‐resolution shell

Crystal	EG00086‐bound	Zn^2+^‐bound
Data collection		
X‐ray source	Diamond I03	ESRF ID23‐2
Space group	C222_1_	C222_1_
Cell constants		
*a*,* b*,* c* (Å)	110.25, 139.52, 106.04	99.39, 105.27, 45.75
α, β, γ (°)	90.00, 90.00, 90.00	90.00, 90.00, 90.00
Resolution (Å)	67.03–1.95 (2.00–1.95)	36.13–1.80 (1.84–1.80)
Completeness (%)	99.5 (97.4)	99.9 (99.8)
Multiplicity	3.4 (3.0)	4.6 (4.5)
*R*_merge_	0.059 (0.223)	0.052 (0.230)
<*I*/σ(*I*)>	10.8 (4.3)	16.5 (5.4)
CC_½_	0.996 (0.944)	0.997 (0.937)
Wilson *B* factor (Å^2^)	23.3	21.7
Refinement		
Number of reflections	59 661	22 736
*R*_work_/*R*_free_	0.204/0.234	0.177/0.217
Mean *B* value, all atoms (Å^2^)	26.0	25.5
RMSDs		
Bond lengths (Å)	0.010	0.016
Bond angles (°)	1.463	1.753
Ramachandran plot (%)		
Favoured, allowed,	96, 4, 0 (chain A)	
outliers	95, 5, 0 (chain B)	96, 4, 0

### Structure of a protein complex of the VEGF‐A_165_ C‐terminus and the NRP2 b1 domain

**Figure 3:**
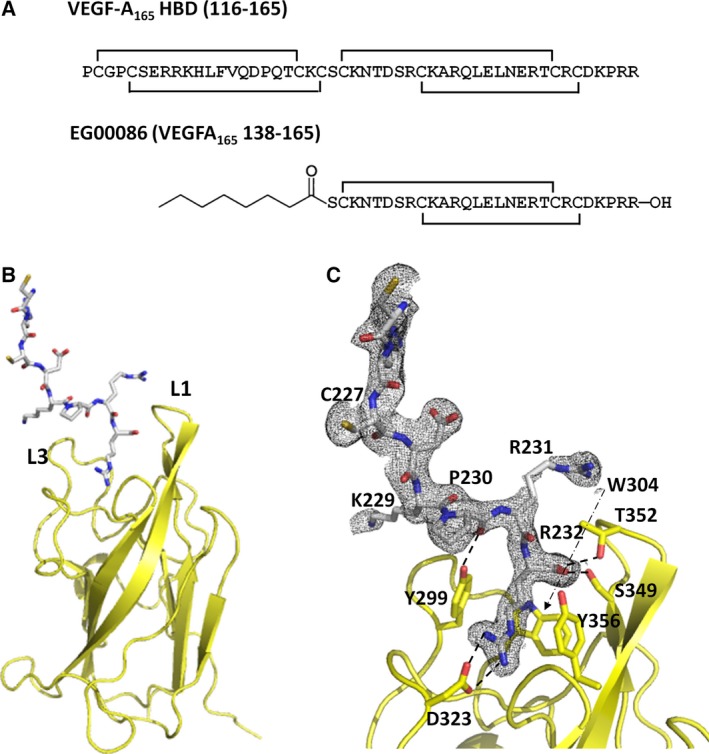
Structure of the VEGF‐A_165_‐derived peptide bound to the NRP2 b1 domain. (A) The amino acid sequences of the heparin binding domain of VEGF‐A_165_ and the related bicyclic peptide EG00086. Disulfide links are indicated. The numbering corresponds to the full‐length VEGF‐A form. (B) A maximum of eight C‐terminal residues of EG00086 (sticks) were detected in the electron density maps of the molecular complex. The peptide binds to the canonical ligand binding site flanked by loops L1 and L3. (C) Detailed view of the interactions between the peptide and NRP2. Relevant residues have been labelled, and the specific hydrogen bonds and charge–charge interactions are indicated by dashed lines. The 2 *F*_o_ – *F*_c_ electron density map (grey mesh) showed significant disorder for the peptide region; in this image, the associated electron density map is contoured at 0.6 σ.

We also obtained a crystal structure of a protein complex of peptide EG00086 (Fig. 3A) bound to the b1 domain of NRP2. Peptide EG00086, corresponding to the C‐terminus of VEGF‐A_165_ (residues 138–165), its binding affinity to NRP1 and NRP2, and its potency to act as inhibitor of VEGF‐promoted cellular adhesion have been previously described 29. The peptide corresponds to the C‐terminal half (one sub‐domain) of the HBD of VEGF‐A_165_. The small sub‐domain comprises short two‐stranded anti‐parallel β‐sheet stabilized by two disulfide bridges. The crystal structure of the complex between the NRP2 b1 domain and EG00086 was refined to 1.95 Å resolution, with four copies of EG00086‐bound NRP2 b1 in the asymmetric unit (Fig. 3 and Table 1). In this structure, the C‐terminal arginine residue of the ligand EG00086 resides in the conserved binding pocket of the NRP2 b1 domain (Fig. 3C). The C‐terminal carboxylate forms hydrogen bonds with Ser349 and Thr352, while the Arg guanidinium group engages in ionic interaction with Asp323 at the base of the binding pocket. The aliphatic portion of the Arg side chain is nestled between Tyr356 and Tyr299, while the hydroxyl group of Tyr299 additionally interacts with EG00086 through a hydrogen bond with the backbone carbonyl oxygen of Pro163 of EG00086 (or residue 230 in a canonical full‐length VEGF‐A sequence, P15692‐1) (Fig. 3C). The NRP2 side chains contributing to tethering of the C‐terminal arginine are conserved in the NRP1 binding pocket 25, 26, 30, and have also been identified as being involved in binding of VEGF‐C within the structure of an NRP2 fusion protein in which the C‐terminus of the NRP2 b1b2 protein product is fused to the last five residues of proteolytically processed VEGF‐C 27. In our structure of the complex between NRP2 b1 domain and the EG00086 peptide, even though there is sufficient space within the crystal packing to accommodate the entire EG00086 molecule, a significant portion of the peptide appeared disordered, and, within the tetrameric structure, only up to eight amino acids of EG00086 per NRP2 binding site were modelled, with some of the side chains not visible in the electron density maps (Fig. 3C). While the C‐terminus of the peptide is well‐tethered to the neuropilin molecule, the rest of the structure displays multiple confirmations, giving rise to crystallographic disorder. Even though the peptide contains disulfide bonds, we were unable to model with confidence the adjacent β‐strand of the β‐hairpin structure. The structure of the complex implies that the main function of the HBD of VEGF may be to provide a specific but flexible connection between the two partner receptors: neuropilin and the VEGF receptor.

Intriguingly, in the EG00086‐bound NRP2 structure reported here and in the recently reported structure of the protein fusion between the NRP2 b1b2 segment and the C‐terminal pentapeptide of proteolytically processed VEGF‐C 27, both VEGF‐A_165_ and VEGF‐C bind to the same NRP2 binding site, and the same set of hydrogen bonds and ionic interactions are involved in binding of the C‐terminal ligand residues. However, we observe differences in the direction of the C‐terminal VEGF‐A_165_‐related peptide between the structure reported here and that reported for the structure of the NRP1 b1–VEGF‐A_165_‐HBD fusion protein 26. However, in both structures, effects of crystal packing on positioning of the VEGF‐A_165_ components of the complexes could not be completely excluded. Our previous isothermal titration calorimetry (ITC) measurements of the *in vitro* interaction between NRP1, NRP2 and EG00086 29, and the crystal structures of the relevant complexes, do not clarify the molecular basis for the differences in biological activity. Considering that qualitatively the same set of key molecular interactions have been identified in all instances, it is possible that additional factors such as the energy of the additional hydrogen bonds within the NRP1/ligand and NRP2/ligand complexes, entropic changes due to side chains or solvent rearrangement within the ligand binding sites, or structural differences and post‐translational modifications elsewhere on the proteins contribute to the perceived differences in the ligand affinities of NRP1 versus NRP2.

### The zinc binding site in the NRP2 b1 domain is remote from the VEGF ligand binding site

**Figure 4:**
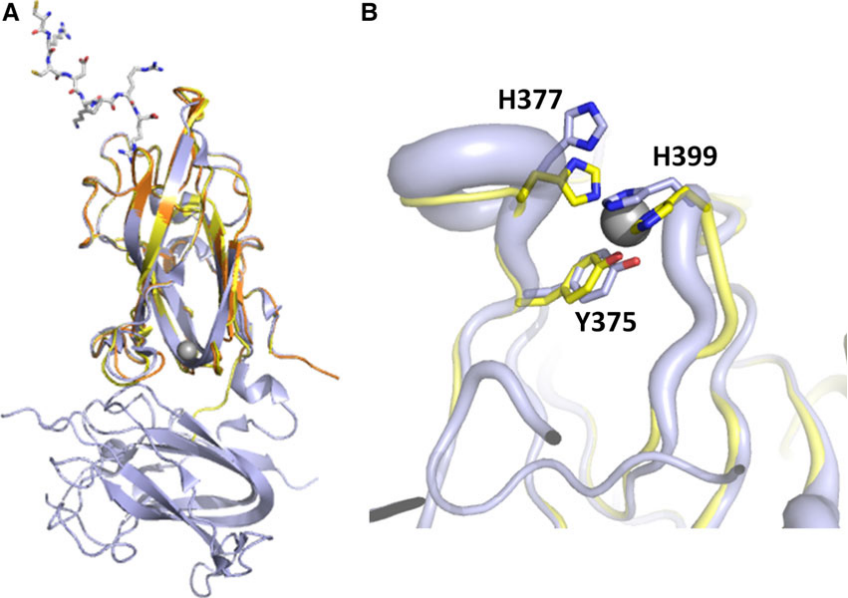
Zn^2+^ ions bind remotely from the peptide ligand binding site on NRP2. (A) Overlay of the two structures reported here (yellow and orange, EG00086 and Zn ion‐bound respectively) with the structure of the NRP2 b1b2 domains (PDB ID 2QQJ) (grey) shows that the main zinc binding site is located near the interface of domains b1 and b2, remote from the ligand binding region. EG00086 is shown using sticks representation, and the zinc ion is indicated by a grey sphere. (B) Close‐up view of the conformational changes associated with Zn^2+^ binding. The zinc‐bound structure shown in yellow is compared to NRP2 b1b2 domains (PDB ID 2QQJ), shown in grey. In this representation, the backbone thickness correlates with the values of the temperature factors for the corresponding backbone atoms.

Overlay of the two structures reported here shows that the main zinc binding site in the NRP2 b1 domain is remote from the VEGF ligand binding site (Fig. 4A), and is unlikely to directly affect ligand binding. Interestingly, although remote from the VEGF binding pocket, zinc ion binding site ‘1’ is located at the edge of the interface between the b1 and b2 domains (Fig. 4A). All neuropilin structures published so far 28, 30, 31 that contain tandem b1b2 domains exhibit an extensive inter‐domain interface, providing fixed orientation of the two constituting domains. Binding of a zinc ion in that region may affect the interface and overall protein conformation and stability. Overlay of our structure with that of the b1b2 domain (PDB ID 2QQJ) shows that, in addition to the adjustments of the side‐chain positions required for ion binding, there is a significant effect on the position of backbone residues and temperature factors in this area (Fig. 4B). There is a localized backbone displacement of 1–2 Å for atoms of NRP2 residues in the regions spanning Tyr375–Asn380 and Ala400–Leu403. Binding of zinc also resulted in reduction of thermal motions of the corresponding atoms (Fig. 4B).

**Figure 5:**
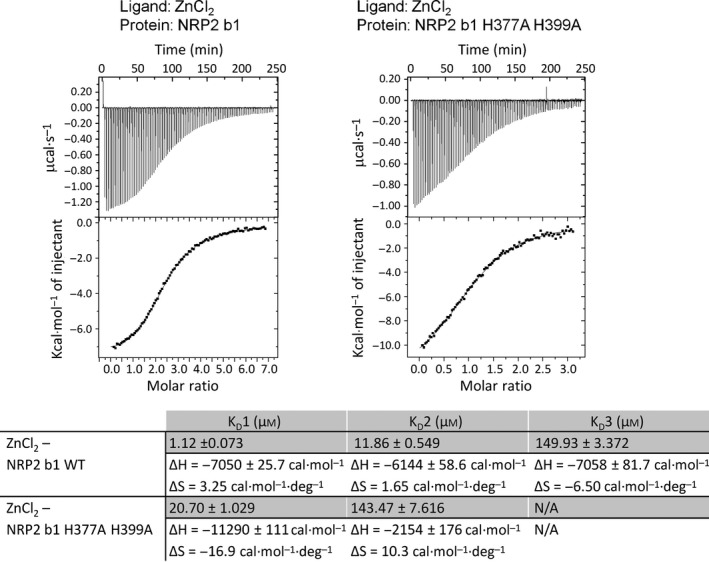
Mutation of residues His377 and His399 leads to a loss of NRP2 b1 domain affinity for zinc. Isothermal titration of ZnCl_2_ and site‐directed mutagenesis were used to confirm the role of residues His377 and His399 in ion binding. The panels on the left show the raw data (top) and the binding isotherm (bottom) for the wild‐type protein, while the panels on the right correspond to the double mutant NRP2 b1b2 protein titrated with ZnCl_2_.

**Figure 6:**
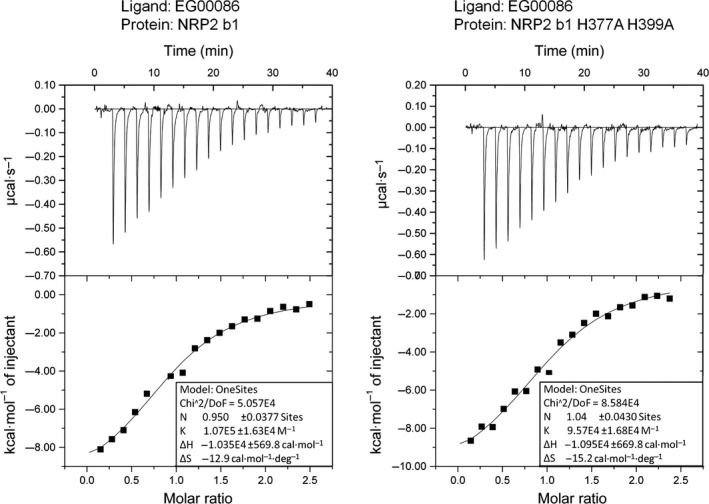
ITC confirmed that mutation of His377 and His399 to Ala did not affect the interaction of the NRP2 b1 domain with EG00086. The panels on the left show the raw data (top) and the binding isotherm (bottom) for the wild‐type protein, while the panels on the right correspond to the double mutant NRP2 b1b2 protein titrated with EG00086.

Zinc ion binding was further investigated using ITC. Consistent with our crystal structure, the binding curve of zinc ion titration into the wild‐type NRP2 b1 domain is fitted with three binding sites (Fig. 5). Our ITC experiments identified the highest‐affinity binding site (site ’1’), with a dissociation constant (*K*_D_) of 1.1 μm. This affinity is expected for a binding site with three side chains coordinating the ion, and is within the range of affinity constants for other physiologically relevant zinc‐binding proteins 32. The micromolar affinity of NRP2 for zinc suggests that, rather than having a structural role, this zinc binding site may have a regulatory function that is exhibited at times of fluctuating physiological zinc concentrations. The other two dissociation constants that were obtained, 11.9 and 149.9 μm, indicate much weaker affinity and may not be of physiological importance. When histidine residues in site ‘1’ were mutated to alanines (His377Ala and His399Ala), the NRP2 b1 domain was still able to bind zinc, but the titration curve was consistent with the presence of two binding sites (Fig. 5) with dissociation constants of 20.7 and 143.5 μm. The two zinc binding sites identified in the His377Ala/His399Ala NRP2 b1 variant have measured affinities comparable to those of the two weaker binding sites identified in wild‐type NRP2 b1. This observation confirms that histidine to alanine mutation of residues 377 and 399 results in abolition of zinc binding site ‘1’. ITC was also used to confirm that the His377Ala/His399Ala mutations did not affect the ligand binding potential of the resultant protein (Fig. 6), with EG00086 association constants of 1.07E5 ± 1.63E4 and 0.96E5 ± 1.68E4 m ^−1^ for the wild‐type and mutant protein, respectively.

### NRP2 thermal stability is decreased in the presence of zinc

**Figure 7:**
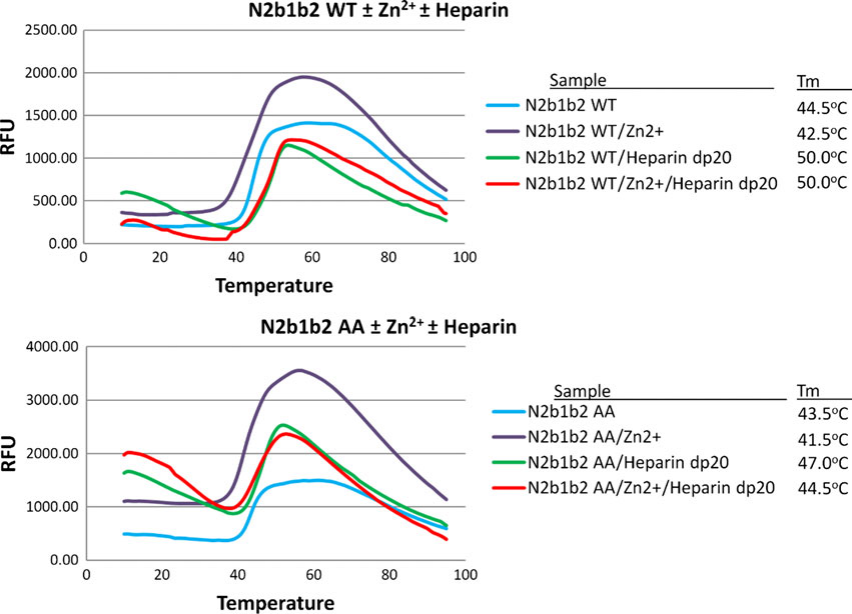
Zinc binding destabilizes NRP2 b1b2 protein. The results of thermofluor assays for the wild‐type protein (top) and the mutant protein (bottom) in the presence of Zn^2+^ and/or heparin demonstrate the protective function of heparin with respect to the protein instability introduced by zinc ion binding.

To further investigate the effect of zinc on NRP2, we performed a fluorescence‐based thermal denaturation assay, which supported our hypothesis that the zinc ion affects the stability of the NRP2 b1b2 protein. Using this rapid, qualitative assessment, we observed that, in the presence of zinc ions, there is a significant negative shift in thermal denaturation curves for all concentrations of Zn^2+^ used. As an example of the effect, Fig. 7 presents thermal denaturation curves showing that the melting temperature (*T*_m_) for the NRP2 b1b2 protein decreased by 2 °C (*T*_m_ for NRP2 b1b2 = 44.5 °C; *T*_m_ for NRP2 b1b2‐Zn^2+^ = 42.5 °C) in the presence of an equimolar amount of zinc. The crystal structure of the b1 domain revealed that Zn^2+^ bound to site ‘1’ bridges the two structural loops that contain the Zn^2+^‐coordinating residues, thereby restricting their flexibility, as determined by the localized reduction in temperature factors. However, in the context of the b1b2 protein, this localized conformational distortion at the inter‐domain interface may contribute to overall loss of thermodynamic stability. The *T*_m_ decreased even further with an increase in zinc ion concentration (data not shown), most likely due to inter‐molecular zinc binding, causing aggregation and faster denaturation. Importantly, not only did the presence of an equimolar concentration of zinc result in NRP2 destabilization, the melting temperature of the histidine to alanine double mutant is itself 1 °C lower than that of wild‐type NRP2 b1b2 (Fig. 7), suggesting that the mutations at site 1 may disrupt the b1/b2 interface at some level, and decrease the overall stability of the variant protein. The effect of zinc in the context of the histidine to alanine double mutant of NRP2 b1b2 is a consequence of zinc ion binding in an inter‐molecular fashion, leading to protein aggregation and denaturation.

### Heparin provides protection from zinc‐induced protein instability

**Figure 8:**
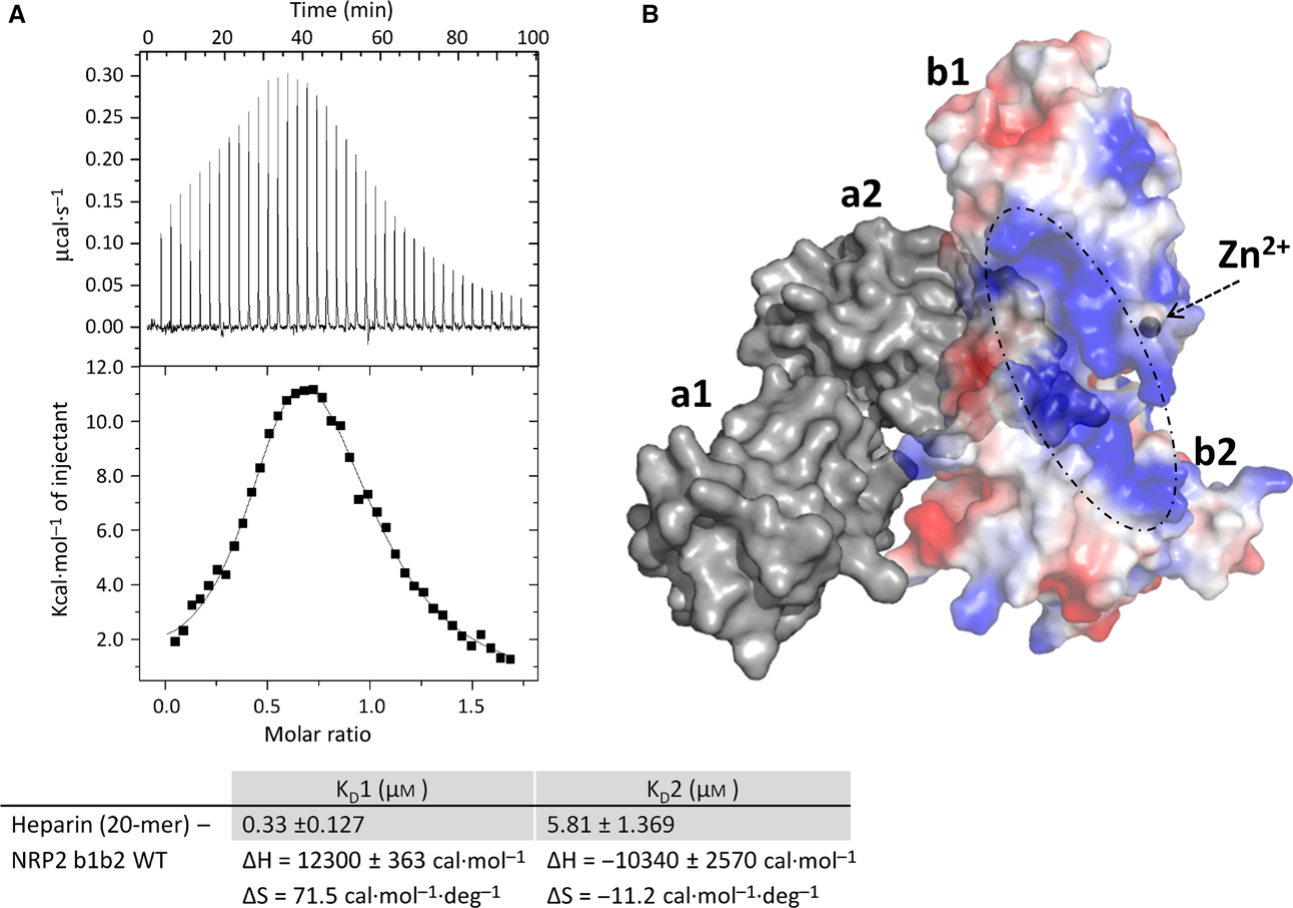
The putative heparin binding region on NRP2 and zinc binding site ‘1’ are close to each other. (A) ITC data for the interaction between heparin and NRP2 b1b2 protein. The top panel shows raw data while the bottom panel shows the binding isotherm obtained by integrating peaks from the top panel. The curve is consistent with the occurrence of two binding events followed by NRP2 dimerization. (B) The NRP2 molecular surface electrostatic potential for the b1b2 domains (PDB ID 2QQJ) is overlaid with the molecular surface for the a1a2b1b2 ectopic region of NRP2 (PDB ID 2QQK), shown in grey. The zinc ion binding site in the NRP2 structure reported here is indicated by a grey sphere, and the region of the positive electrostatic surface potential analogous to the NRP1 heparin binding site is outlined using a dashed line.

Thermal shift assays have also shown that heparin provides protection from zinc‐induced thermal denaturation of NRP2 b1b2 (Fig. 7). It has been reported previously that heparin oligosaccharides bind NRP1 b1b2 and induce protein dimerization 30, and we used ITC to further examine the effect of heparin on the NRP2 b1b2 structure. When heparin oligosaccharides (20‐mer) were titrated into NRP2 b1b2, the ITC binding curve was consistent with two binding events characterized by dissociation constants of 0.33 and 5.81 μm (Fig. 8A). The ITC curve also suggests that, in the initial stages of titration, both binding sites on the heparin molecule (higher‐ and lower‐affinity sites) are occupied by NRP2 b1b2, indicative of a stoichiometry of one molecule of heparin to two molecules of NRP2; but as the heparin concentration increases within the sample chamber, the higher‐affinity binding site is preferentially occupied until all of the NRP2 b1b2 molecules are heparin‐bound in a 1 : 1 stoichiometry. Even though heparin and NRP2 b1b2 form a 1 : 1 binding complex, heparin‐bound NRP2 b1b2 following ITC titration forms a dimer, as suggested by the size‐exclusion chromatography profile. In addition to inducing NRP2 b1b2 dimerization, heparin affects the stability of the NRP2 b1b2 protein, resulting in an increase in the *T*_m_ of heparin‐bound NRP2 b1b2 by 5.5 °C compared to NRP2 b1b2 alone (*T*_m_ = 50 °C and 44.5 °C, respectively) (Fig. 7). To assess the influence of heparin on zinc‐bound NRP2 b1b2 (*T*_m_ = 42.5 °C), we incubated a zinc/NRP2 b1b2 mixture in the presence of heparin for 30 min prior to the thermal shift assay. The presence of heparin effectively reversed the detrimental effect that zinc has on NRP2 b1b2 stability, with a *T*_m_ of 50 °C for heparin‐bound NRP2 b1b2 in the absence and presence of zinc (Fig. 7).

Based on the putative heparin binding site on NRP1 proposed by Vander Kooi *et al*. 30, we identified the corresponding region of the NRP2 b1b2 molecular surface that carries positive charge and thus may potentially be involved in heparin binding (Fig. 8B). Interestingly, the putative heparin binding region and zinc binding site ‘1’ are close to each other. The proximity of the two binding sites may explain, at least in part, the protective effect of heparin with respect to protein destabilization induced by zinc. It is also possible that zinc and oligosaccharides present in the extracellular matrix, although having opposite effects, are both involved in regulating the signalling function of NRP2.

## Discussion

We have reported the first crystal structure of a complex between the peptide corresponding to the C‐terminus of VEGF‐A_165_ and the b1 domain of NRP2. The structure suggests that the atomic details of direct molecular interactions between the b1 protein domain of NRP2 and the C‐terminal ends of VEGF isoforms A and C are similar, as seen from comparisons between the structure reported here (including VEGF‐A_165_) and that of the structure containing the C‐terminus of VEGF‐C 27. The level of structural similarity is somewhat surprising given that NRP1 and NRP2 have distinct physiological preferences for the growth factors, with VEGF‐A_165_ being the main NRP1 ligand, and VEGF‐C signalling primarily through NRP2. Previously, it has been suggested that electrostatic steering provides the mechanism for ligand selectivity between neuropilins and various VEGF isoforms 26. In fact, comparison of the EG00086‐bound NRP2 b1 structure with that of the NRP1 b1–VEGF‐A_165_‐HBD fusion protein shows two significantly different positions of VEGF‐A_165_‐HBD with respect to the neuropilin b1 domain. The backbone of EG00086 is tilted away from the NRP2 molecule, indicating an almost 90° difference in the putative positioning of VEGF‐A_165_‐HBD compared to that suggested by Parker *et al*. for the NRP1 structure 26. The structure reported here supports the hypothesis that VEGF‐A_165_ does not approach the NRP2 binding pocket in the same way as the binding pocket of NRP1 due to differences in the L1 loop of the binding pocket 26. However, it is possible that, in the context of the fusion protein NRP1 b1–VEGF‐A_165_‐HBD and/or under the specific crystallization conditions, VEGF‐A_165_‐HBD was constrained to adopt the observed orientation. It is probable that additional elements of the molecular repertoire, such as glycosylation and other post‐translational modifications (of either NRPs or VEGF molecules), may be important determinants of the ligand recognition mechanism of neuropilins and their physiological function in the context of cell‐surface signalling assemblies.

NRP2 is a key molecule in chemotactic migration of monocyte‐derived dendritic cells, and this function is dependent upon polysialic acid modification of NRP2 33, 34, 35. Polysialylation is a rare post‐translational modification that has best been described in the context of the neural cell adhesion molecule. NRP1 is not modified by polysialylation; however, it has been shown that NRP1 may be modified by chondroitin sulfate attachment to Ser612, and that this modification results in reduced cell migration and invasiveness compared to cells expressing non‐glycosylated NRP1 36, 37. Ser612 is located in the peptide linker region between the b2 domain and the membrane‐proximal c domain of NRP1. Similarly, mass spectrometry analysis of polysialylated NRP2 confirmed that polysialylation modification also occurs in the equivalent region between the NRP2 b2 and c domains 38. NRP2 enhances chemotaxis of dendritic cells towards the chemokine CCL21 in a polysialylation‐dependent fashion, directing dendritic cells to lymphoid organs for activation of naive T cells via antigen presentation.

Our structural studies additionally revealed a new feature of the NRP2 b1 domain structure, namely three Zn^2+^ binding sites, of which the most biologically relevant involves coordination of a Zn^2+^ ion with residues Tyr375, His377 and His399. The location of these residues, remote from the main VEGF binding pocket, suggests that Zn^2+^ binding is unlikely to have a strong influence on NRP2–ligand interaction. ITC experiments have confirmed that mutations of His377 and His399 had no effect on ligand binding. However, mutation of His377 and His399 markedly decreased the thermal stability of the NRP2 b1 domain as revealed by thermal shift assays, an effect that was rescued by heparin binding to the NRP2 b1 domain. We predict that zinc binding to the b1/b2 inter‐domain interface would disrupt NRP2 structure and membrane organization, and possibly affect ligand recognition. However, in our experiments, binding of a model glycan (heparin) to NRP2 supported protein dimerization and protected NRP2 from zinc binding‐induced instability.

The physiological role of Zn^2+^ binding to NRP2 is unclear, but, interestingly, NRP2 has been strongly implicated in maturation of dendritic cells, a process in which zinc homeostasis also plays a crucial role 39, 40, 41. Although the molecular mechanisms through which zinc affects maturation of dendritic cells are not well characterized, it has been shown that expression of molecules of the major histocompatibility complex on the plasma membrane correlates with a decrease in the expression of zinc transporters that move zinc into the cytosol, reduction of intracellular zinc, and an increase in the expression of zinc transporters that transfer ions in the opposite direction across the plasma membrane 39. Our structural identification and *in vitro* investigation of Zn^2+^/NRP2 interactions lay the foundation for further exploration of the effects of zinc homeostasis on neuropilin functions and ligand binding.

## Experimental procedures

### Plasmids

Plasmids expressing recombinant NRP2 domains and VEGF‐A_165_‐HBD have been described previously 42. Wild‐type or mutant pET15b‐TEV:*nrp2‐b1*, pET15b‐TEV:*nrp2‐b1b2* constructs were transformed into Rosetta‐gami 2(DE3)pLysS cells (Novagen, Merck, Darmstadt, Germany), while pET14b:*VEGF*‐*A*_*165*_ *‐HBD* was transformed into SHuffle cells (New England Biolabs, Ipswich, MA, USA).

### Site‐directed mutagenesis

The primers designed to introduce the H377A mutation were 5′‐CCTTGTGGTTTTTGCCAGCCCGGTACACCATC‐3′ and 5′CTGGATGGTGTACCGGGCTGGCAAAAACCAC‐3′. Those designed to introduce the H399A mutation were 5′‐GTCAGCAGTGGAGCGGCGAGCTTGTTCAGAAC‐3′ and 5′‐GGTTCTGAACAAGCTCGCCGCTCCACTGCTG‐3′. PCR was performed using Phusion high‐fidelity DNA polymerase (New England Biolabs) according to the manufacturer's instructions. In each reaction, 10 ng template (pET15b‐TEV:*nrp2‐b1* or pET15b‐TEV:*nrp2‐b1b2*) was added. The PCR product was treated with *Dpn*I at 37 °C for 1 h to cleave the mother template, followed by transformation of *Escherichia coli* DH5α cells (Invitrogen, Thermo Fisher Scientific, Carlsbad, CA, USA). The presence of the doubly mutated gene sequence (His377Ala/His399Ala) was confirmed by DNA sequencing (Source BioScience, Nottingham, UK).

### Expression and purification of recombinant proteins

To over‐express NRP2 b1, NRP2 b1b2 and VEGF‐A_165_‐HBD, 10 mL overnight cultures were transferred into 1 L of LB medium containing the appropriate antibiotics, and cells were grown at 37 °C until the attenuance at 600 nm reached 0.6. All cultures were grown in the presence of 100 ug·ml^−1^ of ampicillin. In addition, tetracyclin at 12.5 ug·ml^−1^ and chloramphenicol at 34 ug·ml^−1^, were used when growing Rosetta‐gami 2 (DE3)pLysS cells. Protein expression was induced by addition of isopropyl‐β‐d‐thiogalactopyranoside at a final concentration of 0.5 mm, and the cells were left overnight at 30 °C on an orbital shaker. Cells were harvested by centrifugation at 3993 ***g*** at 4 °C for 15 min, and washed once for 15 min with ice‐cold buffer A containing 50 mm Tris/HCl, pH 7.9, 300 mm NaCl and 30 mm imidazole. Cells were frozen and stored at −20 °C until used for protein purification.

Cells over‐expressing recombinant NRP2 b1 or b1b2 domains were resuspended in buffer A supplemented with EDTA‐free protease inhibitor (one tablet per 50 mL buffer, Roche) and lysed by sonication. The cell lysate was clarified by centrifugation at 39 100 ***g*** at 4 °C for 30 min. Soluble cell lysate was passed over a pre‐equilibrated nickel ion‐chelating affinity column (HisTrap column, GE Healthcare, Little Chalfont, UK). Tagged proteins were eluted at a flow rate of 1 mL·min^−1^ using a linear gradient of buffer B (50 mm Tris/HCl, pH 7.9, 300 mm NaCl, 600 mm imidazole). Recombinant tobacco etch virus protease was mixed with eluted His‐tagged proteins, followed by dialysis against 4 L of 50 mm Tris/HCl, pH 8.0, 300 mm NaCl, 20 mm imidazole, 0.3 mm l‐cysteine and 3 mm l‐cystine at 4 °C overnight. Following dialysis, protein samples were incubated with pre‐equilibrated Ni‐NTA agarose (Qiagen, Hilden, Germany) for 30 min at 4 °C. Ni‐NTA agarose beads were pelleted by centrifugation at 3700 ***g*** at 4 °C for 10 min. The supernatant was collected and loaded onto a HiTrap Heparin HP column (GE Healthcare) pre‐equilibrated with 50 mm Tris/HCl, pH 7.9, 100 mm NaCl. Target protein was eluted at 1 mL·min^−1^ using a linear gradient of high‐salt buffer comprising 50 mm Tris/HCl, pH 7.9, 2 m NaCl. Eluted recombinant neuropilin protein fractions were pooled, subjected to buffer exchange into 20 mm Tris/HCl, pH 7.9, 50 mm NaCl, and stored at 4 °C.

Cells over‐expressing recombinant VEGF‐A_165_‐HBD were lysed, and His‐tagged proteins were purified using a nickel‐chelating affinity column as described above. Thrombin was mixed with His‐tagged VEGF‐A_165_‐HBD, followed by dialysis against 4 L of 20 mm Tris pH 8.4, 150 mm NaCl, 2.5 mm CaCl_2_, at room temperature overnight. Tagged and untagged proteins were separated by incubation with Ni‐NTA agarose as described above. Untagged proteins were further purified using a HiTrap Heparin (HP) column pre‐equilibrated with 50 mm Tris/HCl, pH 7.9, 100 mm NaCl (GE Healthcare). Target protein was eluted at the flow rate of 1 mL·min^−1^ using a linear gradient of high‐salt buffer containing 50 mm Tris/HCl, pH 7.9, 2 m NaCl. Fractions containing VEGF‐A_165_‐HBD were pooled, concentrated and loaded onto a pre‐equilibrated Superdex‐75 size‐exclusion column (GE Healthcare). VEGF‐A_165_‐HBD was eluted at 1 mL·min^−1^ using buffer containing 50 mm Tris/HCl, pH 7.5, 100 mm NaCl. Samples were pooled, sparated into aliquots, and stored at −20 °C.

### Peptide synthesis

Synthesis of the bicyclic peptide, EG00086, containing the C‐terminal 28 amino acid residues of VEGF‐A_165_ encoded by exons 7 and 8 (equivalent to VEGF‐A_138–165_), was performed as previously described 29.

### X‐ray crystallography

Purified NRP2 b1 domain at 10 mg·mL^−1^ was crystallized in the presence of zinc sulfate. Protein was mixed with mother liquor (25% PEG 550 MME, 0.1 m MES, pH 6.5, 0.01 m ZnSO_4_) at a 1 : 1 ratio, and crystals were grown at 16 °C by the vapour diffusion method. X‐ray diffraction data were obtained at the European Synchrotron Radiation Facility (Grenoble, France) at 1.8 Å resolution, and belonged to space group C2221.

Formation of a complex of the NRP2 b1 domain and peptide EG00086 was confirmed by ITC, after which the sample was collected from the titration cell. The sample contained a 1 : 2 molar ratio of protein to ligand in 20 mm Tris/HCl, pH 7.9, 50 mm NaCl, and NRP2 b1 at a concentration of 10 mg·mL^−1^. The protein complex was mixed with mother liquor (10% PEG 20 000, 0.1 m bicine pH 9.0, 2% dioxane) at a 1 : 1 ratio, and crystals were grown at 16 °C by the vapour diffusion method. X‐ray diffraction data were collected at the Diamond Light Source (Didcot, UK) at 2 Å resolution, and belonged to space group C222_1_ with cell dimensions of *a* = 110.25 Å, *b* = 139.52 Å, *c* = 106.04 Å, α = 90.00°, β = 90.00° and γ = 90.00°.

Data were merged and indexed using iMosflm 43, 44. The structure was solved by molecular replacement using phaser 45 with the apo NRP2 b1 domain (PDB ID 2QQJ) 28. Iterative rounds of refinement were performed using coot 46 and refmac5 47, 48. CC_½_ values were obtained from unmerged data using aimless 49.

### Isothermal titration calorimetry

Wild‐type and mutants of the NRP2 b1 or b1b2 domains were dialysed in 20 mm Tris/HCl, pH 7.9, 50 mm NaCl at 4 °C overnight. Protein was loaded into the cell at 40–200 μm concentration, while ZnCl_2_ was loaded in the syringe at 1.5–5 mm. Zinc titration was performed at 21 °C using 98 injections in a MicroCal iTC200 system (GE Healthcare). EG00086 titration was performed at 15 °C with 19 injections using 40.3 μm protein sample in the cell and 600 μm EG00086 peptide in the syringe. VEGF‐A_165_‐HBD titration was performed at 10 °C with 19 injections, with 80 μm protein sample in the cell and 2.1 mm VEGF‐A_165_‐HBD in the syringe. Titration of heparin oligosaccharides was performed at 21 °C with 39 injections of 300 μm heparin oligosaccharides in the syringe and 36 μm purified NRP2 b1b2 domain in the cell. Data were evaluated using origin version 7.0 software (OriginLab, Northampton, MA, USA) with the ITC plug‐in provided by the manufacturer.

### Thermofluor assay

The wild‐type and mutated NRP2 b1b2 domains (45 μm) were mixed with 0, 10, 25, 45, 80, 150, 200 or 300 μm ZnCl_2_ in 20 mm Tris/HCl, pH 7.9, 50 mm NaCl, at room temperature for 30 min before mixing with SYPRO Orange protein gel stain (final dilution 1 : 1250; Life Technologies, Carlsbad, CA, USA). In the experiments including heparin, 45 μm protein samples were mixed with 45 μm ZnCl_2_ at room temperature for 30 min before addition of 45 μm heparin oligosaccharide dp20 (molecular mass 5750 Da; Iduron, Alderley Edge, UK). The samples were left at 4 °C for 30 min, and then mixed with the dye. The thermofluor assay was performed at 10–95 °C with a 0.5 °C step increment over 75 min using a MyiQ RT‐PCR instrument (Bio‐Rad, Hercules, CA, USA). Experiments were performed in triplicate. Data were evaluated using iq5 software (Bio‐Rad).

### Accession numbers

The the EG00086 and Zn^2+^‐bound structures have been submitted to the Protein Data Bank under accession numbers 5DN2 and 5DQ0, respectively.

## Author contributions

Y.‐C.I.T., S.D., P.F. and I.Z. designed the research. Y.‐C.I.T. performed the majority of the experimental work. T.Y. performed part of the ITC experiments. R.R.R. and C.F. assisted in the refinement. Y.‐C.I.T., I.Z. and S.D. wrote the paper, and all authors revised the manuscript.
